# Feasibility of ecological momentary assessment in measuring physical activity and sedentary behaviour in shift and non-shift workers

**DOI:** 10.1186/s44167-024-00063-7

**Published:** 2024-10-08

**Authors:** Malebogo Monnaatsie, Stuart J.H. Biddle, Tracy Kolbe-Alexander

**Affiliations:** 1 Department of Sport Science, University of Botswana, Gaborone, Botswana; 2 School of Health and Medical Sciences, University of Southern Queensland, Ipswich, Australia; 3 Centre for Health Research, University of Southern Queensland, Springfield, Australia

**Keywords:** Shift work, Ecological momentary assessment (EMA), Prompts, Physical activity, Sedentary time

## Abstract

**Background:**

Previous studies assessing shift workers’ behaviours have mainly used self-report recall questionnaires, however these measures don’t always account for variations in work schedules. Alternative methods that allow for real-time assessments tailored to capture variations in work patterns might provide more accurate measures of physical activity (PA) and sedentary behaviour (SB). Therefore, the aim of this study was to evaluate the feasibility of Ecological Momentary Assessment (EMA), which provides real-time evaluations of PA and SB in shift workers. A secondary aim was to compare shift workers and non-shift worker responses.

**Methods:**

Participants (*n* = 120; 58% female, mean *M*_*age*_=36.0), included 69 shift workers and 51 non-shift workers. After downloading the EMA app, shift workers received either interval-contingent tailored (SW-T) or standardized EMA prompts (SW-S) over 7–10 days, while non-shift workers received standardized prompts (NSW-S) for seven days. Prompts were scheduled five times daily, every three hours. The EMA survey asked participants to report their current activity, including type, duration, and location of physical activity and sitting. Feasibility was assessed by analysing recruitment, retention, and compliance rates (EMA surveys completed) across SW-T, SW-S, and NSW-S groups.

**Results:**

Approximately 78% of invited workers enrolled, and all enrolled workers completed at least one prompt on 4 out 7 days in the NSW-S and 7 out of 10 days in the SW group. Workers who chose not to participate reported unwillingness to travel for meetings (*n* = 14), while others did not respond (*n* = 20). Participants completed an average of 24 surveys per day, each one taking less than 30 s to complete. Overall, 64% of EMA surveys were started and completed. SW-S completed the least prompts (57%), while SW-T and NSW-S completed 64% and 68%, respectively (*p* = 0.90). On average, workers missed 36% EMA surveys which was similar for SW and NSW (*p* = 0.05).

**Conclusion:**

Our study represents one of the few studies that has used EMA in the shift work population with adaptation to shift schedules. The findings showed a modest compliance to EMA. Strategies are needed to enhance compliance rates. However, EMA shows promise for capturing real-time behaviours in shift workers’ natural work environments.

**Supplementary Information:**

The online version contains supplementary material available at 10.1186/s44167-024-00063-7.

## Background

Shift work is defined by work hours outside the regular daytime (06:00–18:00 h) and includes early morning, late afternoon/evening, night, or rotating shifts [1]. In industrialized societies, approximately 15–20% of workers are shift workers in industries like healthcare, manufacturing, mining and transport [2, 3]. Shift work is associated with an increased risk of chronic diseases, including cardiovascular diseases, breast cancer and diabetes [4, 5]. Mechanisms related to shift work and poor health outcomes appear to be multifactorial, including circadian disruption, disturbed sleep, lifestyle behaviours, and psychosocial stress [6]. Previous studies concluded that shift work results in poor sleep quality and dietary patterns [7]. It is also plausible that shift work may impact movement-related behaviours because of rotating work schedules [8, 9]. Exploring the effects of shift work on movement-related behaviours, therefore, can provide a more comprehensive understanding of the association between shift work and health risks.

Regular physical activity, especially moderate-to-vigorous intensity (MVPA), is associated with physiological and psychological benefits and reduced risk of chronic diseases [10, 11]. Replacing sedentary behaviour with any physical activity will reduce the burden of various chronic diseases and produce health benefits [12, 13]. Studying all behaviours within the 24-hour paradigm is crucial for understanding their interconnections in shift work [2]. However, assessing shift workers’ physical activity and sedentary behaviours during shifts, while considering contexts, can be complicated [14]. Therefore, methods that allow for real-time assessments and repeated schedules to capture within-person variations are needed [15]. Ecological momentary assessment (EMA) uses an approach for collecting data in real time that could provide insights into day-to-day health behaviours [15].

EMA is increasingly becoming the method of choice in assessing health behaviour in real-time, overcoming the problems of recall bias [16]. EMA is defined as monitoring or sampling strategies to assess phenomena at that moment they occur in natural settings [17]. The EMA method uses an approach of collecting data in real time including signalling to participants to complete a survey at random or predefined intervals [18]. In EMA studies, participants are instructed to respond to self-report surveys over the course of the day for a short period of time (e.g., weeks and months) using a device such as a mobile phone. It can provide context, type and time, which can be beneficial for providing information on time and shift schedules suitable for developing effective intervention strategies [19]. Contextual information can include intrapersonal, interpersonal, and environmental factors in which behaviour occurs that are integral in understanding outcomes of behaviour [20]. Thus, EMA can be used to understand how work-related factors influence behaviour in the shift work population.

Given the varying work schedules in the shift working population, it creates a challenge to assess their behaviours. Assessing shift workers’ behaviour should be based on waking hours, which may be distributed across all hours of the day because of the work schedules [21]. Therefore, adapting EMA prompts to work schedules can demonstrate how workers experience various health behaviours day-to-day [15]. An increasing number of studies have tested the feasibility of EMA with smartphone applications to measure physical activity and sedentary behaviour in adults and older adults, with compliance rates between 58 and 92% [22–25].

Studies with non-shift work populations have shown that EMA is feasible in assessing health behaviours [23, 25]. For instance, in a workplace study, the response rate was 81.4% for EMA prompts [25]. While EMA feasibility has been assessed in various populations, more studies are needed with shift workers. One shift work study using EMA showed that EMA was feasible [26]. However, all the studies above did not adjust EMA surveys by work schedules, not fully capitalizing on the potential of adjusting EMA prompts accordingly. In addition, the results of a recent systematic review of EMA studies assessing several health outcomes in rotation workers showed inadequate compliance-related information [15]. Because of the nature of work involving shift workers’ unconventional hours, it is important to strategically design EMA studies comparing with non-shift workers in order to determine how adapting EMA to work, non-workdays and individuals affect feasibility. The complexity of this type of work requires accurately documenting workers’ behaviours considering different schedules and tasks [27].

In the present study, the EMA response rates from the two groups of shift workers who receive standardized and tailored prompts were compared to check if tailoring affects feasibility. In addition to feasibility, information on the number of prompts and schedules concerning EMA completion rates is essential for encouraging EMA in real-time interventions. Our primary aim, therefore, was to evaluate the feasibility of EMA in assessing physical activity and sedentary behaviour in shift and non-shift workers. The other objective was to explore how the compliance rate is affected by adapting EMA surveys to shift work schedules versus standardized non-shift workers during work and non-workdays. We hypothesized that EMA will be feasible to use in assessing workers physical activity and sedentary behaviour. We also hypothesized that shift workers will answer more EMA surveys when tailored to their work schedules than when standardized. This is because shift work involves irregular working hours, thus sending EMA surveys at standard times may not be aligned with their work hours.

## Methods and materials

### Study design and participants

Ecological momentary assessments (EMA) interval-contingent experience sampling method [28, 29] was used to assess workers’ physical activity and sedentary behaviour throughout the day. Workers (*N* = 128) were recruited via word of mouth from the research team, participants who recommended the study to other shift workers, flyers handed out at workplaces, and via social media (Facebook and Twitter/*X*). Shift workers employed at various industries on a full-time contract and working rotational schedules (including, early morning and night) were invited. Non-shift workers from various workplaces were included if they were full time and working normal work hours (8/9am-5/6pm) Monday to Friday. Participants were recruited living in and around Brisbane, Ipswich, and Toowoomba, in south-east Queensland, Australia from various workplaces including hospitals, university security, government offices, and the transport industry. The majority of shift workers were nurses and paramedics, and non-shift workers were located in offices. Participants were allocated to groups using a sequential enrollment method [30].

**Fig. 1:**
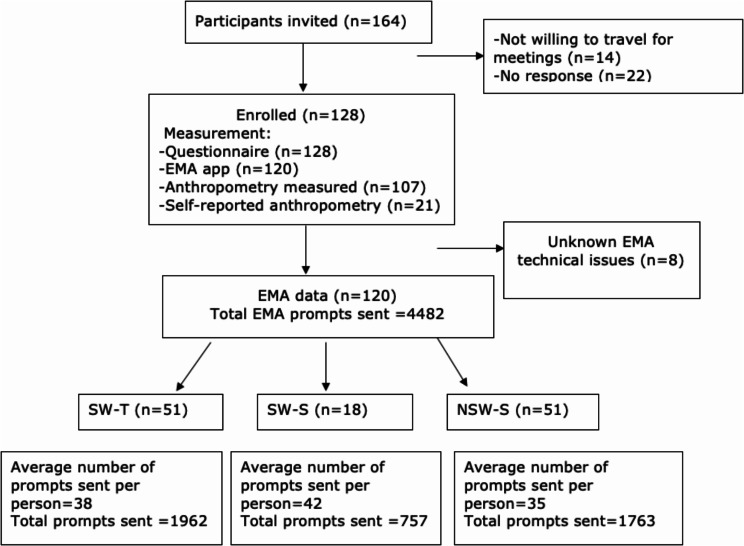
Recruitment and participant flow chart. Legend. HRA: Health risk appraisal, Participants invited represents the number of participants sent emails, EMA prompts sent– the number of EMA surveys successfully sent to mobile phone. EMA prompts per person are number of EMA Surveys sent to each participant. SW-T: shift workers with tailored EMA prompts, SW-S: shift workers who received standardized prompts, NSW-S: non-shift workers with standardised prompts

A flow chart illustrating the enrolment process is in Fig. 1. After receiving an information sheet, participants were invited to an in-person meeting with the researcher. During the meeting participants (1) signed the informed consent form and were sent the link to download the EMA app then given instructions on how to use the EMA app, (2) completed the questionnaires about their demographic characteristics and health risk appraisal, (3) had anthropometric measures taken and, (4) downloaded the EMA app.

### Procedure

Participants completed 7–10 days of EMA one wave of data collection. For shift workers, the number of days was dependent on individual shift schedules to collect EMA data during all work schedules and non-workdays. Non-shift workers participated in the study for seven consecutive days including five workdays and two non-workdays.

The tailored prompts were set according to shift schedule and were different for each participant and 3 h apart. The tailored prompt times were based on anticipated shift schedules and wake-sleep pattern of the shift workers and adjusted when they reported any change of shift schedule. Some of their workdays could fall on weekends. Standardized prompts were sent at the same time of day during work and non-workdays. For example, EMA prompts were scheduled from 10:00 am-10:00 pm during day/morning shifts; from 1:00pm-1:00am during evening and 10:00 pm -10:00 am on night shifts. The standardized EMA prompts were sent between 10:00 AM and 10:00 PM daily. Each EMA survey consisted of five questions, took less than a minute to complete and disappeared after 30 min if unanswered. For non-shift workers the non-workdays were weekend days and working days were Monday to Friday. This avoided shift workers receiving prompts while they were sleeping. Table 1 summarises the EMA prompting schedule.

**Table 1:** EMA prompting patterns and schedule

Group name	Work type	Study period (days)	EMA prompts	Number and timing of prompts per day
SW-T (*n* = 51)	Shift work	7–10	Tailored	5 at times based on work schedules
SW-S (*n* = 18)	Shift work	7–10	Standardized	5 between 10 a.m. and 10 p.m.
NSW-S (*n* = 51)	Non-shift work	7	Standardized	5 between 10 a.m. and 10 p.m.

*Legend.* SW-T: shift workers who received tailored EMA prompts, SW-S: shift workers who received standardized EMA prompts, NSW-S: non-shift workers who received standardized EMA prompts

Upon receiving the EMA prompt on their phones, participants were instructed to stop their current activity, provided it was safe to do so, and complete a 1-minute survey. For all study participants, EMA prompts were sent via the app five times per day at 3-hour intervals similar to other EMA studies [24, 26]. The prompts disappeared after 30 min of no response. At the end of the study, all participants were provided with feedback on their EMA responses and device-based measures. The study obtained approval from the University Human Research Ethics Committee.

#### Ecological momentary assessment

The *SEMA*^*3*^ app link [31] was sent via email for participants to download onto their smartphones. The *SEMA*^*3*^ app was available in both Apple iPhone and android operating systems. The *SEMA*^*3*^ app was developed for conducting EMA surveys and has been used in previous EMA studies [32]. The app allows for delivering surveys at fixed points in time or at fixed time intervals [31]. A Checklist for Reporting EMA Studies (CREMAS) [33] related to our study protocol is presented in supplementary Table 3.

### Measures

#### The EMA survey

**Fig. 2:**
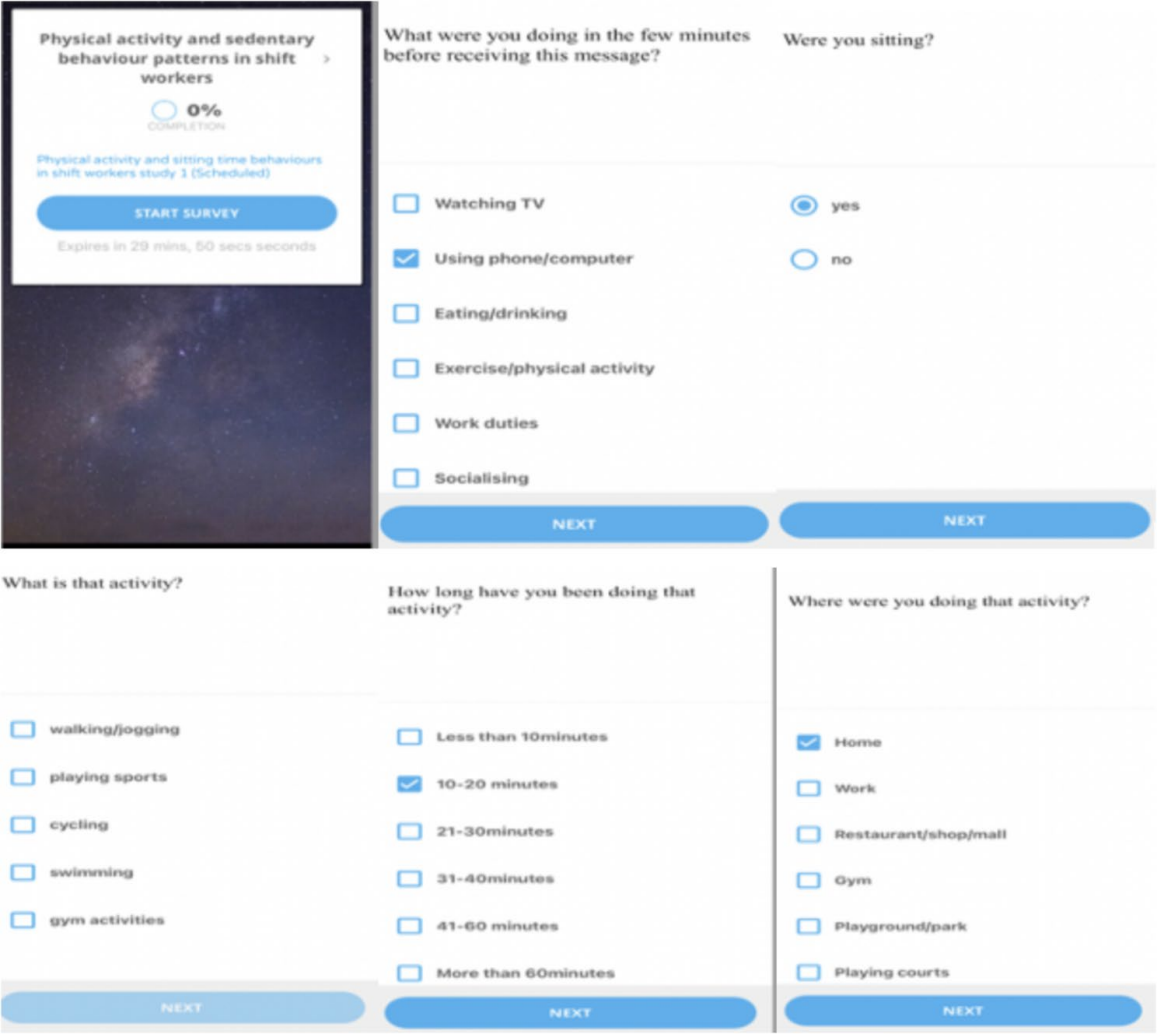
SEMA app screenshots from the researcher phone. The images show how the EMA questions appeared on the mobile phone

The EMA survey assessed participants’ current activity, duration, location, detailed physical activity type (if they chose the physical activity option) and sitting. The survey began with: *“what were you doing in the few minutes before receiving this message?”* Response options included, *“watching television*,* using mobile phone/computer*,* eating/drinking*,* exercise or physical activity*,* work duties*,* socializing*,* driving/travelling*,* and household/garden chores*,* caring for children*,* and other.”* If the participant selected “*watching television or using mobile phone/computer*,* work duties*,* socializing*,* caring for children”*, the next question was a follow-up and asked about sitting. If exercise or physical activity was reported, a follow-up question asked about specific type of exercise or physical activity. If *“other”* was selected, the participants were asked to provide more detailed information on the activity. For each activity reported, participants were asked to report time spent (in minutes) on the activity and their location. The EMA questions were adopted from previous EMA studies assessing physical activity and sedentary behaviour [23, 24]. Survey responses were downloaded from the SEMA website in CSV format and converted to excel files. Figure 2 presents an example of the screen shots from the EMA items.

#### EMA feasibility

The feasibility outcomes were assessed through several key metrics: compliance rate, recruitment and retention rates. Compliance rate was calculated as the proportion of EMA surveys completed relative to those sent, with thresholds set based on prior EMA studies (> 70% for compliance) [33, 34]. Notably, EMA compliance rates in previous research varied widely, ranging from 44 to 96%, with an average of 71% [35]. Recruitment rate was the percentage of eligible participants who agreed to participate and enrol in the study relative to those invited to participate. Retention was defined as the percentage of participants who completed at least one prompt on at least 4 out of 7 days for non-shift workers and 7 out of 10 days for shift workers during data collection [34]. The unanswered EMA surveys were categorized as missed prompts. Survey completion time, from prompt signal to completion, was recorded.

Total EMA responses were calculated over the 7–10 days, adjusted according to participant type (shift worker: 7–10 days; non-shift worker: 7 days). The responses were then examined in relation to various work-related factors such as work versus non-workdays and different shift schedules for shift workers. Feasibility markers were reported for all participants and subgroups (SW-T, SW-S, and NSW-S), assessing the overall viability of EMA implementation. Additionally, associations between prompt responses and participants’ demographic characteristics were explored to understand factors influencing survey completion rates.

#### Anthropometry

Waist circumference measurements (to the nearest 0.5 cm) were taken by placing a tape measure midpoint between the lower border of the rib cage and the iliac crest [36]. Participants’ height was measured to the nearest 0.1 cm using a stadiometer (Seca), with participants standing with their scapula, buttocks and heels resting against a wall, the neck was held in a natural non-stretched position, and the head was held straight. Weight was measured to the nearest 0.1 kg using a digital scale, Seca 803. Participants removed shoes and heavy clothes prior to weighing. Body mass index (BMI) was calculated as weight (kg)/height (m) squared.

#### Health risk appraisal questionnaire

Participants’ age (years), gender (male, female, or prefer not to say), marital status, and shift schedules were reported. The health risk appraisal section asked participants to report their perceived health status by choosing the options ‘poor’, ‘average’, ‘good’ or ‘excellent’. Participants also reported how their work schedule impacted their activities, including leisure time and domestic activities.

### Statistical analyses

The demographic characteristics and feasibility markers were summarised as means and standard deviations for continuous variables, and frequencies and percentages for categorical variables. To test for normality, we used histograms and Shapiro-Wilk tests. We performed a Kruskal-Wallis test to examine the differences in the demographic characteristics of participants in the three work groups (SW-T, SW-S and NSW-S) and EMA compliance between groups. The prediction of answering prompts versus missing them by workdays, non-workdays and shift schedules were examined using multilevel logistic regression analyses [24, 34]. Pearson correlation coefficients were calculated to test associations between EMA compliance rate with age, sex, and BMI. The alpha level was set at 0.05 for all analyses, and all statistics were conducted in SPSS version 27.

## Results

### Participant characteristics

The non-shift workers were significantly older than both the shift work groups (Table 2). Body Mass Index was similar for all groups with an average of 27.9 kg/m^2^, placing participants in the overweight category. Most participants were female (58%), and the majority of non-shift workers were married or living with a partner. Most of the shift workers were nurses and paramedics (66%). The remaining shift workers included security guards, drivers, and manufacturing workers. The non-shift workers were office workers.

Supplementary Table 1 presents EMA-reported activities, with work duties (16.4%) being the most frequent, followed by phone or computer use. Caring for children was the least reported activity (1%). Significant differences in reported physical activity and TV watching were observed across the three work groups, with non-shift workers reporting the highest levels.

**Table 2:** Participant characteristics for each of the three groups

Demographic characteristic	Total (*n* = 120)	SW-T (*n* = 51)	SW-S (*n* = 18)	NSW-S (*n* = 51)	*P*-value
Age (years) mean ± SD	36.0 ± 10.6	31.6 ± 8.5	30.0 ± 8.4	42.1 ± 11.3	0.02
BMI (kg/m^2^) mean ± SD	27.9 ± 5.7	27.0 ± 4.6	27.7 (5.1)	29.0 ± 6.9	0.90
Gender n (%)					0.71
Male	50.0 (41.7)	24.0 (47.0)	8.0 (44.4)	18 (35.3)	
Female	70.0 (58.3)	27.0 (52.9)	10.0 (55.6)	33.0 (64.7)	
Marital status n (%)					0.05
Living with partner	69.0 (59.5)	25.0 (49.0)	9.0 (50.0)	34.0 (66.6)	
Not living with partner	47.0 (40.5)	26.0 (50.9)	9.0 (50.0)	17.0 (33.3)	
Work type n (%) Health care Other Office workers	45.0 (37.5) 29.0 (24.2) 46.0 (38.3)	33.0 (64.7) 18.0 (35.3) -	12.0 (66.7) 6.0 (33.3) -	- 5.0 (9.8) 46.0 (90.2)	

Legend: SW-T: shift workers who received tailored EMA prompts, SW-S: shift workers who received standardized EMA prompts, NSW-S: non-shift workers who received standardized EMA prompts, BMI: Body Mass Index. Health care workers include nurses and paramedics, other represented other various industries including security, drivers, and manufacturing. Living with partner include married and not married but living with partner, Not living with partner include participants who reported that they were single, separated/divorced and widowed. Statistically significant difference (p<0.05) between SW-T, SW-S and NSW-S using Kruskal Wallis test

### EMA feasibility

#### EMA recruitment and retention

Of the 164 workers who were sent emails to participate, 128 volunteered, thus 78% enrollment rate. The reasons for declining participation included being unwilling to travel to the university campuses for the in-person visit, or not responding. All the 128 workers who were enrolled completed the study. However, eight were excluded from the analysis due to unknown EMA technical issues. Thus, 120 participants who completed the study were included in the final analyses.

#### EMA compliance

Table 3 presents workers overall compliance and prompt responses by work and non-workdays. A total of 4482 EMA prompts sent across all study participants. Participants started and responded to 2951 prompts but completed 2900 (64%). On average, each participant received 37 prompts per day. Participants in our study completed an average of 24 surveys per day.

Shift workers who received standard prompts (SW-S) missed about 17 prompts (43%), while SW-T missed 14 prompts (36%). On average NSW-S missed 11 prompts (32%) out of the 35 sent, with the difference between all the three groups being borderline significant (*p* = 0.05). Each survey took an average of 24 s to complete, and this was similar across the three groups (*p* = 0.26). The prevalence of overall completed prompt and missed prompts was similar for the 3 groups (SW-T, SW-S and NSW-S). SW-T answered more prompts during non-workdays than at work. When comparing the shift schedules, SW-T were more likely to answer all the prompts than miss them.

**Table 3:** Summary of EMA overall compliance and prompt responses during work and non-workdays

EMA	Total (*n* = 120)			SW-T (*n* = 51)			SW-S (*n = 18)*			NSW-S (*n* = 51)			*P*-value
	Mean	SD	%	Mean	SD	%	Mean	SD	%	Mean	SD	%	
Completed prompts	23.9	8.7	64.0	24.2	8.7	64.0	24.7	12.9	57.0	23.4	6.9	68.0	0.90
Missed prompts	13.1	8.6	36.0	13.8	9.5	36.0	16.7	9.3	42.9	11.1	6.9	32.0	0.05
Response time (min)	0.40	0.25		0.41	0.33		0.45	0.31		0.34	0.10		0.26
Prompt responses (%)	Non-workday		Workday	Non-workday		Workday	Non-workday		Workday	Non-workday		Workday	
1st Prompt	27.0		24.6	20.5		24.6	29.8		30.3	23.6		26.2	0.64
2nd prompt	22.4		21.7	21.8		22.2	23.7		20.4	21.7		22.4	0.45
3rd prompt	20.7		20.6	21.7		21.4	19.3		19.6	20.9		21.0	0.88
4th prompt	17.1		16.8	18.5		17.8	15.9		14.9	16.9		17.6	0.17
5th prompt	14.3		14.8	17.3		14.0	11.3		14.9	14.3		15.4	0.99

*Legend.* Abbreviations n: total number, SD: standard deviation, min: minutes taken to respond to each survey. SW-T: shift workers with tailored EMA prompts, SW-S: shift workers who received standardized prompts, NSW-S: non-shift workers with standardised prompts. Differences with Kruskal Wallis test between the groups are statistically significant (*p* < 0.05)

**Fig. 3:**
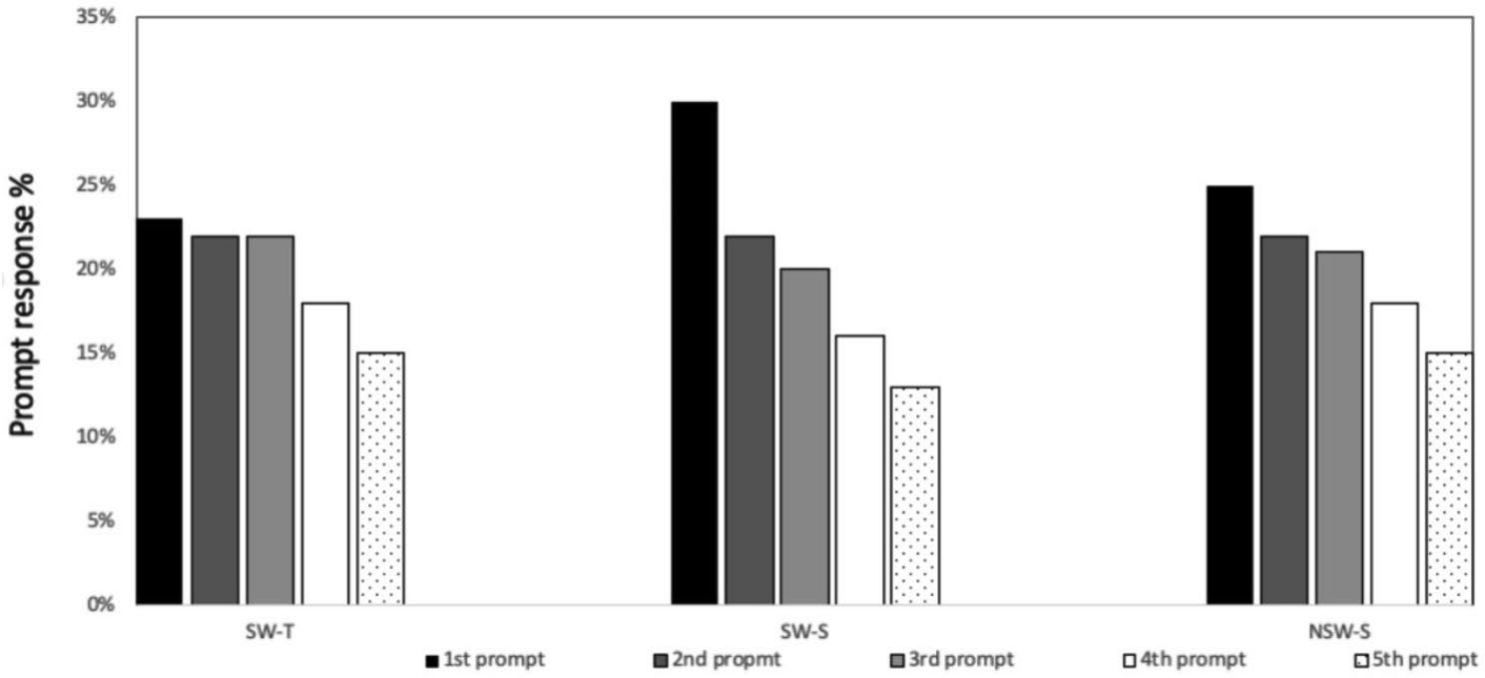
Responses to the five EMA prompts between groups. SW-T: shift workers with tailored EMA prompts, SW-S: shift workers who received standardized prompts, NSW-S: non-shift workers with standardised prompts

Figure 3 shows the completed prompts according to the timing of daily prompts (first to fifth prompt). The most frequently answered was the first prompt of the day (26%), and the fifth was the least answered (14%) in all participants. However, the differences in prompt responses were between prompt 1 and 4 (*p* = 0.04) and prompt 1 and prompt 5 (*p* = 0.01), but no differences in prompt 1, 2 and 3.

SW-T answered more prompts during non-workdays than at work. When comparing the shift schedules, SW-T were more likely to answer all the prompts than miss them. Further, the day and evening shift prompts were frequently answered than night (prompt 3 OR = 0.86, prompt 4 OR = 0.86, and prompt 5 OR = 0.90). SW-S were more likely to answer the first four prompts than miss them during non-workdays than workdays, except the fifth prompt (OR = 0.88). The odds ratios of prompt responses by work, nonwork days and shift schedules are presented in supplementary Table 3. Additionally, SW-S answered more prompts during day and night shifts than evening shift. In contrast, the non-shift work group were likely to answer all the five prompts during weekdays than weekends. EMA compliance was also unrelated to age *(r*=-0.03; *P* = 0.47), gender *(r* = 0.08; *P* = 0.47), BMI (*r* = 0.03; *P* = 0.38), and marital status (*r* = 0.03; *P* = 0.49).

## Discussion

This study assessed EMA feasibility among participants in a work setting. The study aimed for a compliance rate of 70%, but the observed compliance rate was 64%. Although the compliance rate fell below the pre-set goal, a rate of 64% still indicates a substantial level of participant engagement and adherence. The observed compliance rate is within a range that can still yield meaningful insights, given that many EMA studies report comparable compliance rates (58-92%) [23–25].

However, our study findings contrasts with higher compliance rates reported in similar studies of non-shift workers [25]. University employees receiving five daily surveys during workdays achieved an 80% compliance rate [25]. Despite employing a comparable prompt frequency to Weatherson et al. [25], our study achieved a lower overall compliance rate. Conversely, office workers participating in a five-day study with four EMA surveys per day achieved a 58% compliance rate [23]. Several factors may explain the lower-than-expected compliance rate. A systematic review indicated that mobile EMA studies with 1 to 3 prompts per day generally had higher compliance compared to those with more than 3 prompts [29]. However, this evidence is inconsistent, as some studies with fewer prompts reported lower compliance rates [37], while others with more prompts reported higher compliance rates [22, 38].

Notably, our study EMA survey expired after 30 min when unanswered, we did not send reminders. This is potentially contributing to higher compliance rate observed in Weatherson et al.‘s study [25], which aligns with recent findings indicating that frequent reminders can increase overall compliance rates [39]. These results collectively underscore the complex interplay of survey methodology and participant engagement in determining EMA compliance levels across different occupational contexts. Our study compliance rate may have also been affected by lack of monetary incentives. Participants in our study were provided with personalised feedback on their EMA responses and movement. For example, office workers with the higher compliance rate received $20 [23]. In comparison, office workers who did not receive incentives had a lower completion rate [25]. Evidence from a meta-analysis investigating the compliance of EMA showed that giving participants incentives was associated with higher compliance rates [40]. Therefore, compliance rates could be enhanced through the use of incentives and reminders, as the study design itself was robust, evidenced by the fulfillment of 90% of the CREMAS checklist criteria.

More prompts were missed by shift workers who received standard prompts, but the difference was not significant. However, the results should be analysed with caution because of the lower sample size in the group. Previous studies also recommended that EMA prompting be tailored to individual participants’ schedules to increase compliance [28, 41]. Therefore, the method supports estimating physical activity and sedentary behaviour with various predictors of behaviours, such as work, non-work, and shift schedules is possible with EMA.

Our results showed that the first prompt of the day had the highest response rate for shift and non-shift workers. This might be attributable to the time of the day of EMA prompts sent to participants. EMA responses are sometimes able to explain the mental processes [39]. Additionally, response rates were consistent across demographic factors such as age, body mass index, and gender. Similarly, there were no significant differences in age and gender between participants with complete EMA data and those with missing EMA data. Shift workers in our study were younger than non-shift workers. One would expect that the age differences between shift workers and non-shift workers to influence the prompt compliance because it has been shown that younger participants tend to have lower responses to EMA. Rintala et al. concluded that compliance rates are often reduced in younger population due to their busy daily routine or less interest [42]. However, this was not the case in our study as age did not influence compliance rate and this is consistent with the results of Weatherson et al. [25].

Our study had an acceptable enrollment and retention rate. The high retention rate is consistent with studies using similar designs including use of mobile technology and short EMA surveys [42]. EMA studies that allows participants to use their smartphones provides familiarity and convenience [42, 43]. This is evident by our study participants completing each survey in less than a minute (24 s). By comparison, office workers in the Engelen et al. study also took less than one minute to complete each prompt [23]. Given the short time taken to complete the EMA surveys, our study results showed that participant burden may be low.

### Strengths and limitations

Participants provided extensive data for 7–10 days about activities during work and non-workdays. Participants received a total of 4482 prompts throughout the study to all study participants, thus creating a large number of data points per person. In addition, the EMA protocol allowed for evaluation of within-individual variation of work, non-work and shift schedule schedules. These interactions are difficult to obtain when using traditional self-report measures. This study used shift and non-shift work populations, therefore expanding the evidence of use of EMA in both working groups.

The SEMA^3^ application used in this study was still in the research development stage, thus we were not able to find out why some prompts were not delivered to participants. Findings of this study should be considered within the context of this work as a pilot study. The SW-S group had a smaller sample compared to the other groups. Further we did not assess participants’ mood or stressful events. Further, given that the sample included workers in Australia and did not cover all shift work industries, the results cannot be generalised to other countries and all shift workers. EMA prompts set from 10 a.m. to 10 p.m. could have resulted in missing physical activity and sedentary behaviour early morning and late night in non-shift work and shift workers who received standardized prompts. The overall compliance rates could be related to the prompts occurring at inopportune times. It is important to note that mobile EMA may not be appropriate for all industries, for example workers in mining, manufacturing, and retail may not have access to their phones during work hours. Therefore, these findings should be interpreted with caution. Acceptability assessments could have provided a more comprehensive understanding of how the study may translate into practice and whether it is likely to be well-received by the intended population. Future research should incorporate acceptability evaluations to ensure participant engagement and identify potential issues.

## Conclusions

This study represents one of the few studies that used EMA in the shift work population. The current study showed modest compliance rate but higher retention and enrollment rates. This suggest that EMA is feasible to assess physical activity and sedentary behaviour in workers, but more work is needed on how to increase compliance when tailoring prompts to shift work schedules. EMA provides real-time physical activity and sedentary behaviour data to reach large populations affordably [44]. Repeated monitoring of behaviours with EMA may also result in awareness of behaviour and result in altering of behaviour, for example breaking up patterns of sitting [41]. The insights gained and the successful adaptation of EMA protocols highlight the study’s practical viability and provide a foundation for refining methods in future research. The use of tailoring prompts can inform future health promotion programs using mobile apps on when to deliver prompts to improve behaviours based on the days and times of the day when they answered more prompts.

## Electronic supplementary material

Below is the link to the electronic supplementary material.

Supplementary Material 1

## Data Availability

No datasets were generated or analysed during the current study.

## References

[1] Cheng P, Drake C. Shift work disorder. Neurol Clin. 2019. 10.1016/j.ncl.2019.03.003. 31256790 10.1016/j.ncl.2019.03.003

[2] Crowther ME, Ferguson SA, Reynolds AC. Longitudinal studies of sleep, physical activity and nutritional intake in shift workers: a scoping review. Sleep Med Rev. 2022. 10.1016/j.smrv.2022.101612. 35248964 10.1016/j.smrv.2022.101612

[3] Sweileh WM. Analysis and mapping of global research publications on shift work (2012–2021). J Occup Med Toxicol. 2022. 10.1186/s12995-022-00364-0. 36514070 10.1186/s12995-022-00364-0 PMC9747264

[4] Torquati L, Mielke GI, Brown WJ, Kolbe-Alexander T. Shift work and the risk of cardiovascular disease. A systematic review and meta-analysis including dose-response relationship. Scand J Work Environ Health. 2018. 10.5271/sjweh.3700. 29247501 10.5271/sjweh.3700

[5] Wu QJ, Sun H, Wen ZY, Zhang M, Wang HY, He XH, Jiang YT, Zhao YH. Shift work and health outcomes: an umbrella review of systematic reviews and meta-analyses of epidemiological studies. J Clin Sleep Med. 2022. 10.5664/jcsm.9642. 34473048 10.5664/jcsm.9642 PMC8804985

[6] Cable J, Schernhammer E, Hanlon EC, et al. Sleep and circadian rhythms: pillars of health-a keystone Symposia report. Ann N Y Acad Sci. 2021;1506(1):18–34. 10.1111/nyas.14661. 34341993 10.1111/nyas.14661 PMC8688158

[7] Hulsegge G, Proper KI, Loef B, Paagman H, Anema JR, van Mechelen W. The mediating role of lifestyle in the relationship between shift work, obesity and diabetes. Int Arch Occup Environ Health. 2021;94(6):1287–95. 10.1007/s00420-021-01662-6. 33704584 10.1007/s00420-021-01662-6 PMC8292292

[8] Feng T, Booth BM, Baldwin-Rodríguez B, Osorno F, Narayanan S. A multimodal analysis of physical activity, sleep, and work shift in nurses with wearable sensor data. Sci Rep. 2021. 10.1038/s41598-021-87029-w. 33888731 10.1038/s41598-021-87029-w PMC8062546

[9] Proper KI, van de Langenberg D, Rodenburg W, Vermeulen RCH, van der Beek AJ, van Steeg H, van Kerkhof LWM. The relationship between Shift Work and metabolic risk factors: a systematic review of Longitudinal studies. Am J Prev Med. 2016. 10.1016/j.amepre.2015.11.013. 26810355 10.1016/j.amepre.2015.11.013

[10] Baumann H, Heuel L, Bischoff LL, Wollesen B. mHealth interventions to reduce stress in healthcare workers (fitcor): study protocol for a randomized controlled trial. Trials. 2023. 10.1186/s13063-023-07182-7. 36869368 10.1186/s13063-023-07182-7 PMC9985281

[11] Bull FC, Al-Ansari SS, Biddle S, Borodulin K, Buman MP, Cardon G, Carty C, Chaput JP, Chastin S, Chou R, Dempsey PC, DiPietro L, Ekelund U, Firth J, Friedenreich CM, Garcia L, Gichu M, Jago R, Katzmarzyk PT, Lambert E, Leitzmann M, Milton K, Ortega FB, Ranasinghe C, Stamatakis E, Tiedemann A, Troiano RP, van der Ploeg HP, Wari V, Willumsen JF. World Health Organization 2020 guidelines on physical activity and sedentary behaviour. Br J Sports Med. 2020. 10.1136/bjsports-2020-102955. 33239355 10.1136/bjsports-2020-102601 PMC7719912

[12] Park JH, Moon JH, Kim HJ, Kong MH, Oh YH. Sedentary lifestyle: overview of updated evidence of potential health risks. Korean J Fam Med. 2020. 10.4082/kjfm.20.0165. 33242381 10.4082/kjfm.20.0165 PMC7700832

[13] Matthews CE, Moore SC, Sampson J, Blair A, Xiao Q, Keadle SK, et al. Mortality benefits for replacing sitting time with different physical activities. Med Sci Sports Exerc. 2015;47:1833–40. 10.1249/MSS.0000000000000621. 10.1249/MSS.0000000000000621 PMC4515413 25628179

[14] Chappel SE, Aisbett B, Considine J, et al. Measuring nurses’ on-shift physical activity and sedentary time by accelerometry or heart rate monitoring: a descriptive case study illustrating the importance of context. JASSB. 202310.1186/s44167-023-00036-214. 10.1186/s44167-023-00036-2 PMC11960232 40217432

[15] Asare BY, Robinson S, Kwasnicka D, Powell D. Application of Ecological Momentary Assessment in studies with Rotation workers in the resources and related construction sectors: a systematic review. Saf Health Work. 2023;15. 10.1016/j.shaw.2022.10.004. 10.1016/j.shaw.2022.10.004 PMC10024174 36941930

[16] Degroote L, DeSmet A, De Bourdeaudhuij I, Van Dyck D, Crombez G. Content validity and methodological considerations in ecological momentary assessment studies on physical activity and sedentary behaviour: a systematic review. Int J Behav Nutr Phys Act. 2020;16. 10.1186/s12966-020-00932-9. 10.1186/s12966-020-00932-9 PMC7063739 32151251

[17] Shiffman S. Designing protocols for ecological momentary assessment. In: Stone AA, Shiffman S, Atienza AA, Nebeling L, editors. The science of real-time data capture: self-reports in health research. New York: Oxford University Press. 2007;19:27–53.

[18] Romanzini CLP, Romanzini M, Batista MB, Barbosa CCL, Shigaki GB, Dunton G, Mason T, Ronque ERV. Methodology used in ecological momentary Assessment studies about sedentary behavior in children, adolescents, and adults: systematic review using the Checklist for reporting ecological momentary Assessment studies. J Med Internet Res. 2019;18. 10.2196/11967. 10.2196/11967 PMC6540725 31094349

[19] Bedard C, King-Dowling S, McDonald M, Dunton G, Cairney J, Kwan M. Understanding Environmental and Contextual influences of Physical Activity during First-Year University: the feasibility of using Ecological Momentary Assessment in the MovingU study. JMIR Public Health Surveill. 201710.2196/publichealth.7010. 19. 28566264 10.2196/publichealth.7010 PMC5471502

[20] Perske O, Keller J, Kale D, Asare BY, Schneider V, Powell D, Naughton F, Ten Hoor G, Verboon P, Kwasnicka D. Understanding health behaviours in context: a systematic review and meta-analysis of ecological momentary assessment studies of five key health behaviours. Health Psychol Rev. 2022. 10.1080/17437199.2022.211225820. 10.1080/17437199.2022.2112258 PMC9704370 35975950

[21] Torquati L, Mielke GI, Brown WJ, Burton NW, Kolbe-Alexander TL. Shift work and poor Mental Health: a Meta-analysis of Longitudinal studies. Am J Public Health. 201910.2105/AJPH.2019.305278 21. 31536404 10.2105/AJPH.2019.305278 PMC6775929

[22] Dunton GF, Liao Y, Kawabata K, Intille S. Momentary assessment of adults’ physical activity and sedentary behavior: feasibility and validity. Front Psychol. 201210.3389/fpsyg.2012.00260.39 23. 22866046 10.3389/fpsyg.2012.00260 PMC3408114

[23] Engelen L, Chau JY, Burks-Young S, Bauman A. Application of ecological momentary assessment in workplace health evaluation. Health Promot J Austr. 2016. 10.1071/HE16043. 40 24. 27596817 10.1071/HE16043

[24] Maher JP, Rebar AL, Dunton GF. Ecological Momentary Assessment is a feasible and valid Methodological Tool to measure older adults’ physical activity and sedentary behavior. Front Psychol. 201810.3389/fpsyg.2018.01485.41 25. 30158891 10.3389/fpsyg.2018.01485 PMC6104625

[25] Weatherson K, Yun L, Wunderlich K, Puterman E, Faulkner G. Application of an ecological momentary Assessment Protocol in a workplace intervention: assessing compliance, Criterion Validity, and reactivity. J Phys Act Health. 201910.1123/jpah.2019-0152.42 26. 31541068 10.1123/jpah.2019-0152

[26] Rebar AL, Alfrey KL, Gardner B, Vandelanotte C. Health behaviours of Australian fly-in, fly-out workers and partners during on-shift and off-shift days: an ecological momentary assessment study. BMJ Open. 2018;43. 10.1136/bmjopen-2018-023631. 10.1136/bmjopen-2018-023631 PMC6318530 30580269

[27] Kolbe-Alexander TL, Gomersall S, Clark B, Torquati L, Pavey T, Brown WJ. A hard day’s night: time use in shift workers. BMC Public Health. 2019;452. 10.1186/s12889-019-6766-5. 10.1186/s12889-019-6766-5 PMC6546613 31159755

[28] Asare BY, Robinson S, Kwasnicka D, Powell D. Application of Ecological Momentary Assessment in studies with Rotation workers in the resources and related construction sectors: a systematic review. Saf Health Work. 2023;14(1):10–6. 10.1016/j.shaw.2022.10.004. 36941930 10.1016/j.shaw.2022.10.004 PMC10024174

[29] Himmelstein PH, Woods WC, Wright AGC. A comparison of signal- and event-contingent ambulatory assessment of interpersonal behavior and affect in social situations Psychol assess. 2019; 10.1037/pas0000718. 30. 10.1037/pas0000718 PMC6591090 30958026

[30] Chen Z, Zhao Y, Cui Y, Kowalski J. Methodology and application of adaptive and sequential approaches in contemporary clinical trials. J Probab Stat. 2012;2012:527351.

[31] Koval P, Hinton J, Dozo N, Gleeson J, Alvarez M, Harrison A, Vu D, Susanto R, Jayaputera G, Sinnott R. Sema3: Smartphone ecological momentary assessment, version 3. Computer software. 2019. https://www.sema3.com.31.

[32] Maes I, Mertens L, Poppe L, Crombez G, Vetrovsky T, Van Dyck D. The variability of emotions, physical complaints, intention, and self-efficacy: an ecological momentary assessment study in older adults. PeerJ. 202210.7717/peerj.13234. 32. 35611175 10.7717/peerj.13234 PMC9124457

[33] Hartson KR, Huntington-Moskos L, Sears CG, Genova G, Mathis C, Ford W, Rhodes RE. Use of Electronic Ecological Momentary Assessment methodologies in physical activity, sedentary behavior, and Sleep Research in Young adults: systematic review. J Med Internet Res. 2023;25:e46783. 10.2196/46783.29. 37384367 10.2196/46783 PMC10365632

[34] Nam S, Dunton GF, Ordway MR, et al. Feasibility and acceptability of intensive, real-time biobehavioral data collection using ecological momentary assessment, salivary biomarkers, and accelerometers among middle-aged African americans. Res Nurs Health. 202010.1002/nur.22068 35. 32856310 10.1002/nur.22068 PMC8985242

[35] Williams MT, Lewthwaite H, Fraysse F, Gajewska A, Ignatavicius J, Ferrar K. Compliance with Mobile Ecological Momentary Assessment of Self-reported health-related behaviors and psychological constructs in adults: systematic review and Meta-analysis. J Med Internet Res. 2021. 10.2196/17023. 33656451 10.2196/17023 PMC7970161

[36] Ross R, Neeland IJ, Yamashita S, Shai I, Seidell J, Magni P, Santos RD, Arsenault B, Cuevas A, Hu FB, Griffin BA. Waist circumference as a vital sign in clinical practice: a Consensus Statement from the IAS and ICCR Working Group on visceral obesity. Nat Reviews Endocrinol. 2020;16:3. 10.1038/s41574-019-0310-7 PMC7027970 32020062

[37] Jones A, Remmerswaal D, Verveer I, Robinson E, Franken IHA, Wen CKF, Field M. Compliance with ecological momentary assessment protocols in substance users: a meta-analysis. Addiction. 2019;114(4):609–19. 10.1111/add.14503. 30461120 10.1111/add.14503 PMC6492133

[38] Ono M, Schneider S, Junghaenel DU, Stone AA. What affects the completion of ecological momentary assessments in chronic Pain Research? An Individual Patient Data Meta-Analysis. J Med Internet Res. 2019;21(2):e11398. 10.2196/11398. Published 2019 Feb 5. 30720437 10.2196/11398 PMC6379815

[39] Markowski KL, Smith JA, Gauthier GR, Harcey SR. JMIR Form Res. 2021;5(9):e31421. 10.2196/3142137. Patterns of Missing Data With Ecological Momentary Assessment Among People Who Use Drugs: Feasibility Study Using Pilot Study Data. 10.2196/31421 PMC8501406 34464327

[40] Wrzus C, Neubauer AB. Ecological Momentary Assessment: A Meta-Analysis on Designs, Samples, and Compliance Across Research Fields. Assessment. 2023; 10.1177/10731911211067538.39 10.1177/10731911211067538 PMC9999286 35016567

[41] Elavsky S, Klocek A, Knapova L, Smahelova M, Smahel D, Cimler R, Kuhnova J. Feasibility of real-time Behavior Monitoring Via Mobile Technology in Czech adults aged 50 years and above: 12-Week study with Ecological Momentary Assessment. JMIR Aging. 2021;39. 10.2196/15220. 10.2196/15220 PMC8663589 34757317

[42] Rintala A, Wampers M, Myin-Germeys I, Viechtbauer W. Response compliance and predictors thereof in studies using the experience sampling method. Psychol Assess. 2019. 10.1037/pas0000662.57-40. 30394762 10.1037/pas0000662

[43] Dauber S, Beacham A, West A, Devkota J, Barrie K, Thrul J. Ecological momentary assessment of heavy episodic drinking in the early postpartum period: a feasibility study. Drug Alcohol Depend Rep. 2023;38. 10.1016/j.dadr.2023.100146. 10.1016/j.dadr.2023.100146 PMC10066518 37012980

[44] Lau SCL, Connor LT, King AA, Baum CM. Multimodal Ambulatory monitoring of Daily Activity and Health-related symptoms in Community-Dwelling survivors of Stroke: feasibility, acceptability, and Validity. Arch Phys Med Rehabil. 2022. 10.1016/j.apmr.2022.06.002. 35780826 10.1016/j.apmr.2022.06.002 PMC10338086

