# Supplementary material for: Feasibility of ecological momentary assessment in measuring physical activity and sedentary behaviour in shift and non-shift workers
Source: J Act Sedentary Sleep Behav. 2024 Oct 8;3:24. doi: 10.1186/s44167-024-00063-7 (PMC11960382; doi:10.1186/s44167-024-00063-7)
Supplement: Supplementary file 1 — Supplementary Material 1 [file 44167_2024_63_MOESM1_ESM.docx]

**EMA feasibility supplementary file**

**Table 1.** EMA reported activities by occupation group [n (%)]

| **Survey question** | **Total**  ***(N=120)*** | **SW-T**  ***(N=51)*** | **SW-S**  ***(N=18)*** | **NSW-S**  ***(N=51)*** |
| --- | --- | --- | --- | --- |
| Watching TV  Using phone/computer  Eating/drinking | 365* (8.0)  461 (10.1)  262 (5.7) | 140 (7.2)  136 (6.9)  105 (5.4) | 69 (9.1)  81 (10.7)  35 (4.6) | 156 (8.3)  244 (13.0)  122 (6.5) |
| Physical activity/exercise | 171* (3.7) | 43 (2.2) | 39 (5.2) | 89 (4.8) |
| Work duties  Socialising  Sleeping  Caring for children  Chores  Travelling | 750 (16.4)  137 (3.0)  141 (3.1)  48 (1.0)  231 (5.0)  223 (4.9) | 311 (21.0)  58 (3.0)  68 (3.5)  18 (0.9)  78 (4.0)  98 (5.0) | 70 (9.2)  16 (2.1)  31 (4.1)  4 (0.1)  36 (4.8)  45 (5.9) | 269 (14.4)  63 (3.4)  42 (2.2)  26 (1.4)  117 (6.3)  80 (4.3) |
| Others | 166 (3.6) | 84 (4.3) | 30 (4.0) | 52 (2.8) |

*Legend:* SW-T: shift workers with tailored EMA prompts, SW-S: shift workers who received standardized prompts, NSW-S: non-shift workers with standardised prompts. Differences with Kruskal Wallis test between the groups are statistically significant represented with * (p<0.05).

**Table 2.** CREMAS CHECKLIST

Feasibility of ecological momentary assessment in measuring physical activity and sedentary behaviour in shift and non-shift workers

| Item | Description |  |
| --- | --- | --- |
|  |  |  |
| 1. Title | Include ecological momentary assessment in title and key words | Yes |
| 1. Rationale | Briefly introduce the concept of EMA and provide reasons for utilizing EMA for this study or topic of interests | Yes |
| 1. Training | Indicate if, and by what methods, training of participants for EMA protocol was used | Yes, App use |
| 1. Technology | Describe what technology, if any, was used. | Yes |
| 1. Wave duration | State the number of waves for the study | One wave |
| 1. Monitoring period | State the number of days each wave of the study lasted, and how many weekdays versus weekend days | Yes, 7-10 days |
| 1. Prompting design | Indicate the prompting strategy used for the study (eg, event-based, interval-based, or a combination of the two). If using interval-based strategy, indicate what type of schedule is | Yes, Fixed |
| 1. Prompt frequency | Intended frequency of prompts per day. | Five times per day |
| 1. Design features | Describe any design feature to address potential sources of bias (eg, reactivity) or participant burden (eg, EMA questions appearing in different orders) | No |
| 1. Attrition | Indicate participant attrition throughout the study | Yes |
| 1. Prompt delivery | Report number of EMA prompts that were planned to be delivered. If possible, also report the number of EMA prompts that were actually received by participants and indicate reasons for why prompts were not sent out | Yes |
| 1. Latency | Report the amount of time from prompt signal to answering of prompt | Yes |
| 1. Compliance rate | Report total answered EMA prompts across all subjects and the average number of EMA prompts answered per person. | Yes |
| 1. Missing data | Report whether EMA compliance is related to demographic or time-varying variables | Yes |
| 1. Limitations | Discuss limitations of the study, taking into account sources of potential bias when using EMA methods | Yes |
| 1. Conclusions | Provide a general interpretation of results and discuss the benefits of using EMA | Yes |

**Table 3**. Prompt responses by work, nonwork days and shift schedules

|  | **SW-T**  **OR (95% CI)** | | | **SW-S**  **OR (95% CI)** | | | **NSW-S**  **OR (95% CI)** |
| --- | --- | --- | --- | --- | --- | --- | --- |
| **Prompt** | **Non-workday** | **Dayshift** | **Nightshift** | **Non-workday** | **Dayshift** | **Nightshift** | **Workday (weekday)** |
| 1^st^ | 1.54* (1.01, 2.33) | 1.36 (0.86, 2.17) | 1.47 (0.88, 2.49) | 1.18 (0.54, 2.57) | 1.28 (0.54, 3.04) | 1.44 (0.61, 3.38) | 1.29 (0.94, 1.75) |
| 2^nd^ | 2.09* (1.35, 3.26) | 1.33 (0.82, 2.14) | 1.46 (0.85, 2.92) | 1.96 (0.72, 5.34) | 1.87 (0.63, 5.58) | 2.25 (0.76, 6.64) | 1.30 (0.96, 1.78) |
| 3^rd^ | 1.98* (1.28, 3.07) | 1.21 (0.76, 1.92) | 0.86 (0.48, 1.52) | 2.56 (0.74, 8.84) | 3.26 (0.89, 11.9) | 3.26 (0.89, 12.0) | 1.42* (1.03, 1.96) |
| 4^th^ | 2.23* (1.38, 3.59) | 1.83* (1.09, 3.06) | 0.86 (0.44, 1.71) | 2.02 (0.58, 7.03) | 2.20 (0.58, 8.39) | 2.40 (0.64, 9.02) | 1.39* (1.00, 1.93) |
| 5^th^ | 2.33* (1.41, 3.83) | 1.67 (0.95, 2.91) | 0.90 (0.44, 1.86) | 0.88 (0.31, 2.50) | 1.02 (0.32, 3.28) | 1.56 (0.52, 4.73) | 1.64*(1.10, 2.47) |

*Legend.* SW-T: shift workers with tailored EMA prompts, SW-S: shift workers who received standardized prompts, NSW-S: non-shift workers with standardised prompts, OR=odds ratio, CI: Confidence interval.

Note: OR to predict the likelihood of answering prompt 1-5 than missing it as the reference category. For SW-T and SW-S prompt responses during workdays and evening shifts were used as comparison groups, while weekend was used for NSW-S. *p<0.05
